# Leisure-time physical activity, daily sitting time, and risk of mortality among CVD patients: a prospective cohort study

**DOI:** 10.3389/fcvm.2025.1672135

**Published:** 2025-10-02

**Authors:** Changnan Xu, Kai Jin, Yike Sun, Mengbiao Cai, Jian Zhu, Yi Yang

**Affiliations:** ^1^ Physical Education Department, Nanjing University of Aeronautics and Astronautics, Nanjing, China; ^2^ Department of Physical Education, Southeast University, Nanjing, China; ^3^ Sports Coaching College, Beijing Sport University, Beijing, China; ^4^ College of Physical Education, Qingdao Hengxing University of Science and Technology, Qingdao, China

**Keywords:** cardiovascular disease, leisure-time physical activity, daily sitting time, mortality, cohort study

## Abstract

**Background:**

Cardiovascular disease (CVD) is a leading cause of mortality globally. While leisure time physical activity (LTPA) and daily sitting time (DST) are known modulators of health, their combined impact on mortality risk specifically among CVD patients warrants further investigation. This study aimed to examine the independent and joint associations of LTPA and DST with mortality in a nationally representative sample of US adults with CVD.

**Methods:**

This prospective cohort study included 2,335 adults with CVD (weighted mean age 61.56 years; 57.12% male) from the National Health and Nutrition Examination Survey (NHANES) 2007–2018, followed for a median of 5.75 years. LTPA was categorized as inactive (0 min/week), insufficiently active (1–<150 min/week), and sufficiently active (≥150 min/week). DST was categorized as 0–<6, 6–<8, and ≥8 h/days. Mortality data were obtained through linkage to the National Death Index until December 31, 2019. Cox proportional hazards models estimated hazard ratios (HRs) and 95% confidence intervals (CIs), adjusting for covariates.

**Results:**

Over 14,139 person-years, 552 deaths (197 CVD-related) occurred. Sufficient LTPA (≥150 min/week) was associated with reduced risks of all-cause (HR: 0.45, 95% CI: 0.31–0.66, *P* < 0.001), CVD (HR: 0.51, 95% CI: 0.25–1.02, *P* = 0.058), and non-CVD mortality (HR: 0.43, 95% CI: 0.28–0.65, *P* < 0.001). Per 60 min/week increase in weekly LTPA was associated with 10% (HR: 0.90, 95% CI: 0.85–0.95) lower risk of all-cause mortality, 12% (HR: 0.88, 95% CI: 0.79–0.97) lower risk of CVD mortality, and 9% (HR: 0.91, 95% CI: 0.84–0.98) lower risk of non-CVD mortality, respectively. DST ≥8 h/days increased risks of all-cause (HR: 1.89, 95% CI: 1.50–2.37, *P* < 0.001), CVD (HR: 2.22, 95% CI: 1.51–3.26, *P* < 0.001), and non- CVD mortality (HR: 1.75, 95% CI: 1.29–2.36, *P* < 0.001) compared to <6 h/days. Combined analyses showed the lowest mortality risk in sufficiently active individuals with DST <6 h/days (all-cause HR: 0.27, 95% CI: 0.18–0.41, *P* < 0.001).

**Conclusion:**

This study suggests that increasing leisure-time physical activity and reducing sedentary time may have potential benefits for reducing mortality in patients with CVD.

## Introduction

Cardiovascular disease (CVD) stands as the primary cause of morbidity and mortality globally, imposing a substantial burden on individuals, healthcare systems, and societies (1). Effective secondary prevention strategies are crucial for improving the prognosis and quality of life for those with CVD. Among these strategies, lifestyle adjustments play a pivotal role, with physical activity serving as a cornerstone (2). Leisure-time physical activity (LTPA), encompassing activities performed during discretionary time, has been consistently linked to numerous health benefits, including reduced risk of premature mortality in the general population and in individuals with chronic conditions such as cancer and type 2 diabetes (2–4). Recognizing these benefits, public health recommendations, like the 2018 Physical Activity Guidelines for Americans, suggest that adults engage in at least 150 min of physical activity per week, or an equivalent mix, to attain significant health advantages. However, a substantial number of adults worldwide fail to meet these fundamental activity guidelines, perpetuating the persistent public health issue.

In addition to concerns regarding physical inactivity, there is a growing acknowledgment of sedentary behavior as a distinct health hazard. Sedentary behavior is defined as any waking behavior with an energy expenditure of ≤1.5 metabolic equivalents (METs) while in a sitting, reclining, or lying position (5). Common examples of sedentary behaviors include watching television, using computers, and prolonged sitting during work (6, 7). Modern lifestyles, characterized by increased screen time, reliance on motorized transportation, and sedentary occupations, have resulted in unprecedented levels of daily sitting time (DST) among populations (8). Prolonged sitting has been identified as an independent risk factor for overall mortality, CVD mortality, and cancer mortality (9). Importantly, sedentary behavior is not simply the lack of physical activity; individuals can adhere to physical activity guidelines while still accumulating significant sedentary time, a phenomenon sometimes termed the “active couch potato” (10).

The individual impacts of LTPA and DST on health outcomes are well-documented. However, further investigation is needed to understand the interaction between these factors, particularly in individuals with established CVD. Previous studies have explored the combined effects of physical activity and sedentary behavior on mortality in the general population (11), as well as in specific patient groups such as cancer survivors (3, 12) and stroke survivors (13). These studies suggest that high levels of physical activity may partially mitigate the risks associated with prolonged sitting. Nonetheless, there is a lack of comprehensive data on these combined effects within a diverse cohort of CVD patients from a nationally representative sample. A deeper understanding of how the interplay between LTPA and DST influences mortality risk in this vulnerable population can provide more nuanced public health recommendations and inform targeted interventions (14).

This study aimed to investigate the separate and combined associations of LTPA and DST with all-cause, CVD, and non-CVD mortality in a nationally representative sample of US adults with pre-existing CVD. It was hypothesized that higher LTPA levels and lower DST would be independently linked to reduced mortality risk, and that individuals with high LTPA and low DST levels would exhibit the lowest mortality risk.

## Methods

### Study participants

NHANES, a nationally representative program of surveys designed to assess the health and nutritional status of adults and children in US, examined approximately 5,000 non-institutional civilians each year. NHANES was approved by the National Center for Health Statistics (NCHS) Ethics Review Board and had obtained informed consents from participants.

A total of 32,016 non-pregnant adults (20–79 years) from NHANES 2007–2018 cycles were selected for eligibility screening. We excluded 133 subjects with missing mortality information, 42 subjects without LTPA data, and 156 subjects without DST data, with 31,705 subjects left. Moreover, 29,370 participants free of CVD and 647 subjects with missing covariates were excluded. Those who answered yes to the questions “Has a doctor or other health professional ever told you that you had congestive heart failure, coronary heart disease, angina/angina pectoris, heart attack, or stroke?” were identified as CVD patients. Finally, the current prospective cohort study comprised 2,335 CVD patients, and the flow of eligibility screening was shown in Supplementary Figure S1.

### Leisure-time physical activity and daily sitting time

LTPA and DST were collected with the Global Physical Activity Questionnaire. Participants were asked to report the frequency and duration of moderate and vigorous LTPA over the past week with questions “In a typical week, on how many days do you do vigorous/moderate-intensity sports, fitness or recreational activities?” and “How much time do you spend doing vigorous/moderate-intensity sports, fitness or recreational activities on a typical day?”. In this study, the amount of LTPA was determined by the total weekly minutes of moderate-to-vigorous activity reported by participants, calculated as: total LTPA = moderate activity (minutes per week) + 2*vigorous activity (min per week) (13). The time spent in LTPA as summarized and participants were classified as inactive (0 min/week), insufficiently active (1–<150 min/week), and sufficiently active (≥150 min/week), according to the 2018 Physical Guidelines for Americans (15). Participants were also asked “How much time do you usually spend sitting on a typical day?”. Sitting time was defined as daily time spent sitting at work, at home, getting to and from places, or with friends, including time spent sitting at a desk, traveling in a car or bus, reading, playing cards, watching television, or using a computer. In accordance with recent studies, DST was categorized into 3 groups: 0–<6, 6–<8, and ≥8 h/days (3, 13).

### Outcome assessment

The outcomes of interest in our study were all-cause, CVD, and non-CVD mortality, which were identified through linkage to the National Death Index until December 31, 2019. Causes of death had been ascertained according to the International Classification of Diseases, Tenth Revision (ICD-10) by the NCHS. In the current prospective cohort study, CVD deaths were defined as deaths attributed to heart disease or cerebrovascular disease (ICD codes I00-I09, I11, I13, I20-I51, I60-I69). Non-CVD deaths included deaths attributable to malignant neoplasms, chronic lower respiratory diseases, accidents, diabetes, influenza and pneumonia, nephritis, nephrotic syndrome and nephrosis, and all other causes. Follow-up duration was defined as the interval from the mobile examination center date to the date of death or December 31, 2019, whichever occurred first.

### Covariates

Demographic, socioeconomic, lifestyle information was collected with a computer-assisted personal interview system by trained interviewers. Covariates were selected based on established associations with physical activity, sedentary behavior, and mortality, including age, sex, race/ethnicity, obesity, marital status, education, income, smoking, alcohol use, hypertension, diabetes, and hypercholesterolemia (13). Race/ethnicity was categorized as non-Hispanic white, non-Hispanic black, Mexican American, and others. Body mass index (BMI) was calculated as weight (kg) divided by height (m) squared. Those with BMI ≥ 30.0 kg/m^2^ were considered as obese. Marital status was categorized as married, single (widowed, divorced, separated, never married), and living with partner. Self-reported education attainment was grouped as under high school, high school, and above high school. Family poverty-income ratio (PIR), which was calculated by dividing family (or individual) income by the poverty guidelines specific to the survey year, was applied to measure income status. Current smoking was defined as smoking cigarettes when surveyed. Current drinking was defined as consuming any type of alcoholic beverage in the past 12 months. Blood pressure measurements were collected by examiners with mercury sphygmomanometers. Hypertension was defined as systolic blood pressure ≥140 mmHg, diastolic blood pressure ≥90 mmHg, physician-diagnosed hypertension, or currently taking prescribed medicine for hypertension. Diabetes was defined as fasting plasma glucose (FPG) ≥ 126 mg/dl, glycated hemoglobin A1c (HbA1c) ≥6.5%, self-reported physician-diagnosed diabetes, use of insulin or oral hypoglycemic medication. Hypercholesterolemia was defined as total cholesterol (TC) ≥240 mg/dl, physician-diagnosed hypercholesterolemia, or currently taking prescribed medicine for hypercholesterolemia.

### Statistical methods

Taking the complex, multistage, and probability sampling design of NHANES into account, we applied sampling weights and sample design variables in analyses. We selected the MEC exam weight (wtmec2yr) and combined the cycles with the recommended methods (https://wwwn.cdc.gov/nchs/nhanes/tutorials/Weighting.aspx). Continuous variables were expressed with weighted means and standard errors (SEs), and categorical variables were expressed with numbers and weighted percentages. Population characteristics across LTPA and DST groups were compared with linear regression for continuous variables and logistic regression for categorical variables.

The log-rank test and Kaplan–Meier (K-M) survival analysis were used to investigate differences in survival probabilities across LTPA and DST groups. Cox proportional hazards regression model was applied to examine the association between LTPA and DST and mortality risk. The proportional hazards assumptions were tested with Schoenfeld residuals method, and no violation was observed (Supplementary Table S1). In model 1, we adjusted age (<60, ≥ 60 years), sex (male, female), and race/ethnicity (non-Hispanic white, others). In model 2, we further adjusted for obesity (yes, no), marital status (married, others), family poverty-income ratio (<3.5, ≥3.5), education attainment (above high school, high school and below), current smoking (yes, no), current drinking (yes, no), hypertension (yes, no), diabetes (yes, no), and hypercholesterolemia (yes, no). To examine whether the associations between LTPA and DST and mortality risk differed by age, sex, race/ethnicity, obesity, marital status, education attainment, family PIR, smoking, alcohol drinking, hypertension, diabetes, and hypercholesterolemia, we performed stratified and interaction analyses. To investigate the joint effect, participants were categorized based on PA and DST, KM survival analysis as well as multivariable Cox proportional hazards regression models were used to estimate mortality risk. We performed the following sensitivity analyses to evaluate the robustness of our results. We excluded deaths that occurred within the first one year of follow-up. In addition, we calculated an assessment of potential residual confounding with *E*-values, defined as the minimum strength of association on the HR scale that an unmeasured confounder would need to have with both the exposure and the outcome to fully explain away the observed exposure-outcome association, conditional on the measured covariates (16, 17).

All statistical analyses were conducted with R version 4.4.2 (The R Foundation for Statistical Computing, Vienna, Austria). Two-sided *P*-values <0.05 were considered as statistical significance.

## Results

### Population characteristics

A total of 2,335 CVD patients (weighted mean age 61.56 years and 57.12% male) were comprised in this prospective cohort study (Table 1). Those with sufficiently active LTPA levels were more likely to be male, non-Hispanic white, married, well-educated, had better income status, current non-smokers, current drinkers, and less likely to have obesity, hypertension, and diabetes (all *P* < 0.05) (Table 1). More daily sitting time was found in those with inactive LTPA levels (*P* < 0.001) (Table 1). Those with higher DST levels were more likely to be older, obesity, non-Hispanic white, diabetic, and physically inactive (all *P* < 0.05) (Supplementary Table S2).

**Table 1:** Characteristics of the study population according to the levels of leisure-time physical activity.

Variable^a^	All (*n* = 2,335)	Levels of leisure-time physical activity			
		Physical inactive (*n* = 1,572)	Insufficiently active (*n* = 290)	Sufficiently active (*n* = 473)	*P*-value^b^
Age, years	61.56 (0.33)	61.69 (0.40)	61.40 (0.73)	61.34 (0.76)	0.89
BMI, kg/m^2^	31.55 (0.22)	32.19 (0.27)	32.22 (0.51)	29.55 (0.38)	<0.001
Male, *n* (%)	1,361 (57.12)	870 (54.25)	164 (52.21)	327 (67.15)	<0.001
Obesity, *n* (%)	1,203 (53.61)	845 (57.18)	162 (59.65)	196 (41.21)	<0.001
Race/ethnicity, *n* (%)					<0.001
Non-Hispanic white	1,114 (71.31)	735 (68.25)	139 (74.83)	240 (77.13)	
Non-Hispanic black	590 (12.61)	408 (13.92)	85 (14.22)	97 (8.40)	
Mexican American	243 (4.76)	169 (5.11)	24 (3.85)	50 (4.34)	
Others	388 (11.33)	260 (12.72)	42 (7.10)	86 (10.13)	
Marital status, *n* (%)					<0.001
Married	1,200 (57.09)	756 (51.86)	157 (61.12)	287 (68.14)	
Single	1,012 (37.04)	735 (41.64)	118 (35.63)	159 (26.14)	
Living with a partner	123 (5.87)	81 (6.50)	15 (3.24)	27 (5.72)	
Education attainment, *n* (%)					<0.001
Under high school	746 (21.68)	593 (27.11)	65 (13.33)	88 (12.48)	
High school	607 (27.83)	415 (29.65)	77 (30.03)	115 (21.99)	
Above high school	982 (50.49)	564 (43.24)	148 (56.64)	270 (65.53)	
Family PIR, *n* (%)					<0.001
<1.3	980 (29.95)	750 (36.17)	98 (21.79)	132 (18.67)	
1.3–<3.5	881 (39.17)	606 (43.53)	115 (34.03)	160 (30.91)	
≥3.5	474 (30.88)	216 (20.30)	77 (44.18)	181 (50.41)	
Current smoking, *n* (%)	618 (25.92)	459 (30.73)	68 (19.86)	91 (17.02)	<0.001
Current drinking, *n* (%)	1,277 (60.30)	786 (54.77)	182 (68.53)	309 (69.81)	<0.001
Hypertension, *n* (%)	1,812 (73.58)	1,262 (77.85)	213 (68.26)	337 (65.69)	<0.001
Diabetes, *n* (%)	1,011 (38.46)	741 (44.22)	105 (32.65)	165 (27.04)	<0.001
Hypercholesterolemia, *n* (%)	1,637 (72.01)	1,111 (73.04)	204 (67.73)	322 (71.73)	0.34
Daily sitting time, hours/day, *n* (%)					<0.001
<6	1,034 (41.66)	635 (36.52)	139 (47.59)	260 (51.45)	
6–<8	441 (18.99)	309 (18.20)	50 (17.91)	82 (21.61)	
≥8	860 (39.35)	628 (45.28)	101 (34.50)	131 (26.94)	

^a^ Continuous variables were expressed as weighted means and standard errors and categorical variables were expressed as numbers and weighted percentages. The sums of percentages may not reach 100%, owing to the rounding of decimals.

^b^ Characteristics across levels of leisure-time physical activity were compared with linear regression for continuous variables and logistic regression for categorical variables.

BMI, body mass index; PIR, poverty-income ratio.

### Associations between LTPA and DST and risk of mortality among CVD patients

**Figure 1:**
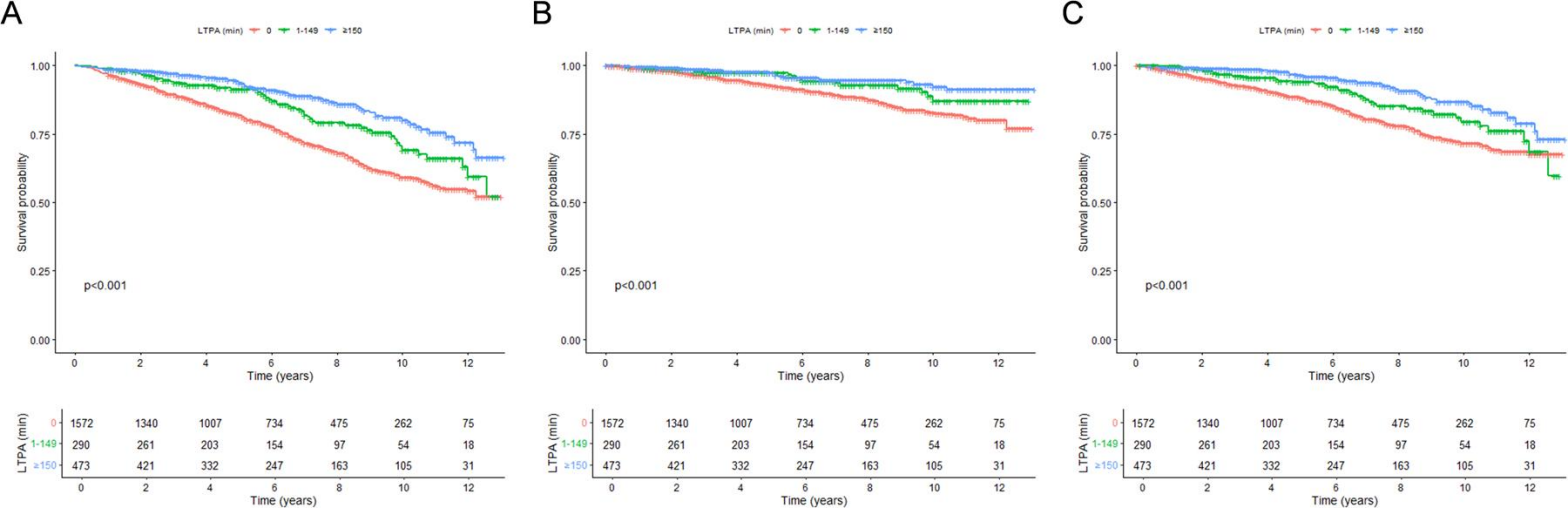
Kaplan-Meier survival curves for mortality, stratified by levels of leisure-time physical activity. **(A)** All-cause mortality; **(B)** CVD mortality; **(C)** non-CVD mortality. LTPA, leisure-time physical activity.

**Figure 2:**
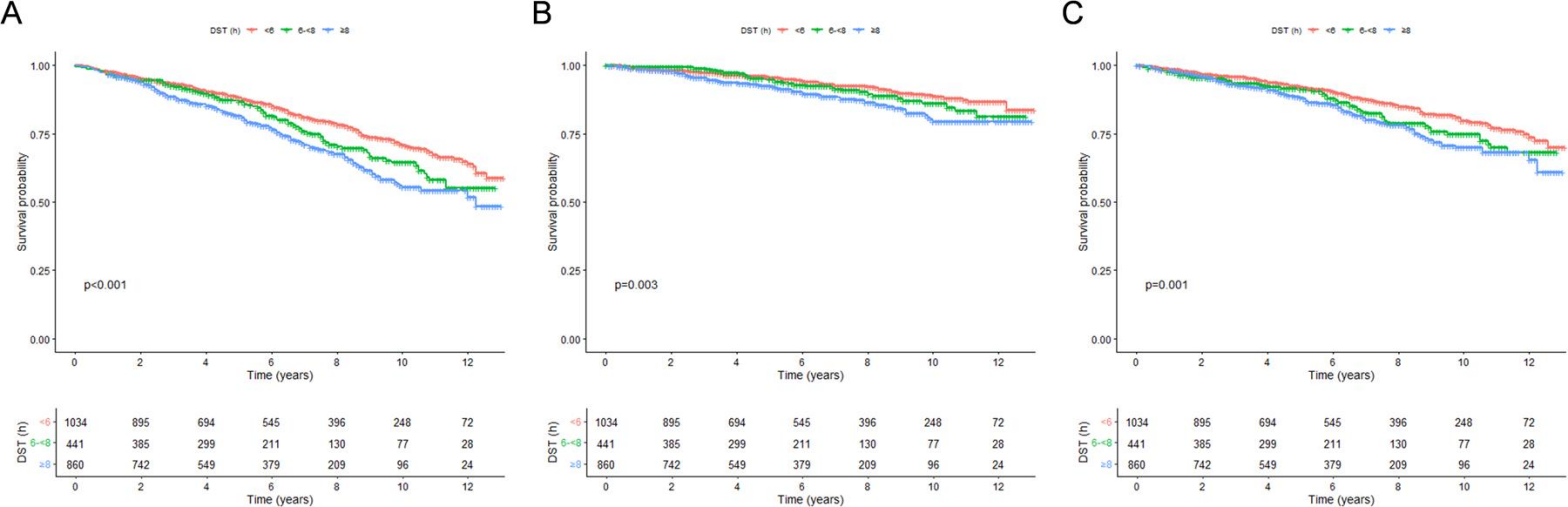
Kaplan-Meier survival curves for mortality, stratified by levels of daily sitting time. **(A)** All-cause mortality; **(B)** CVD mortality; **(C)** non-CVD mortality. DST, daily sitting time.

During 14,139 person-years of follow-up (median follow-up, 5.75 years), a total of 552 deaths, of which 197 died of CVD, were ascertained. The Kaplan–Meier (K-M) survival analysis demonstrated significant differences in all-cause, CVD, and non-CVD mortality rates across levels of LTPA and DST (all log-rank tests *P* *<* 0.001) (Figures 1,2). After adjusting for all covariates, per hour increase in weekly LTPA was associated with 10% (HR: 0.90, 95% CI: 0.85–0.95) lower risk of all-cause mortality, 12% (HR: 0.88, 95% CI: 0.79–0.97) lower risk of CVD mortality, and 9% (HR: 0.91, 95% CI: 0.84–0.98) lower risk of non-CVD mortality, respectively (Table 2). Moreover, compared with those with DST < 6 h/days, subjects with DST ≥8 h/days were faced with 7% (HR: 1.07, 95% CI: 1.04–1.11) greater risk of all-cause mortality, 9% (HR: 1.09, 95% CI: 1.05–1.13) greater risk of CVD mortality, and 6% (HR: 1.06, 95% CI: 1.02–1.10) greater risk of non-CVD mortality, respectively (Table 2). To investigate whether the associations between LTPA and DST and mortality risk differed by age, sex, race/ethnicity, obesity, marital status, education attainment, family PIR, smoking, alcohol drinking, hypertension, diabetes, and hypercholesterolemia, we performed stratified and interaction analyses and observed no significant interactions, with all *P*-interaction >0.05 (Supplementary Tables S3–S8).

**Table 2:** The associations of leisure-time physical activity and daily sitting time with all-cause, CVD, and non-CVD mortality among CVD patients.

Outcome	Event/No.	Crude model		Model 1^a^		Model 2^b^	
		HR (95% CI)	*P*-value	HR (95% CI)	*P*-value	HR (95% CI)	*P*-value
Leisure-time physical activity							
All-cause mortality							
LTPA groups (min/wk)							
0	434/1,572	Reference		Reference		Reference	
1–<150	56/290	0.62 (0.46–0.84)	0.002	0.56 (0.42–0.76)	<0.001	0.74 (0.55–1.01)	0.055
≥150	62/473	0.34 (0.23–0.50)	<0.001	0.32 (0.22–0.47)	<0.001	0.45 (0.31–0.66)	<0.001
Continuous (per 1 h)	552/2,335	0.85 (0.80–0.92)	<0.001	0.84 (0.78–0.91)	<0.001	0.90 (0.85–0.95)	0.001
CVD mortality							
LTPA groups (min/wk)							
0	157/1,572	Reference		Reference		Reference	
1–<150	18/290	0.70 (0.41–1.18)	0.182	0.64 (0.38–1.11)	0.110	0.86 (0.50–1.47)	0.574
≥150	22/473	0.39 (0.20–0.74)	0.004	0.35 (0.18–0.68)	0.002	0.51 (0.25–1.02)	0.058
Continuous (per 1 h)	197/2,335	0.84 (0.75–0.93)	0.001	0.82 (0.73–0.92)	0.001	0.88 (0.79–0.97)	0.012
Non-CVD mortality							
LTPA groups (min/wk)							
0	277/1,572	Reference		Reference		Reference	
1–<150	38/290	0.59 (0.43–0.81)	0.001	0.52 (0.38–0.72)	<0.001	0.68 (0.49–0.95)	0.025
≥150	40/473	0.32 (0.20–0.50)	<0.001	0.30 (0.19–0.48)	<0.001	0.43 (0.28–0.65)	<0.001
Continuous (per 1 h)	355/2,335	0.86 (0.79–0.94)	0.001	0.85 (0.78–0.94)	0.001	0.91 (0.84–0.98)	0.008
Daily sitting time							
All-cause mortality							
DST groups (h/days)							
<6	214/1,034	Reference		Reference		Reference	
6–<8	107/441	1.31 (0.94–1.82)	0.108	1.28 (0.93–1.77)	0.135	1.24 (0.91–1.69)	0.166
≥8	231/860	1.83 (1.45–2.31)	<0.001	1.79 (1.40–2.28)	<0.001	1.89 (1.50–2.37)	<0.001
Continuous (per 1 h)	552/2,335	1.07 (1.04–1.11)	<0.001	1.07 (1.03–1.10)	<0.001	1.07 (1.04–1.11)	<0.001
CVD mortality							
DST groups (h/days)							
<6	75/1,034	Reference		Reference		Reference	
6–<8	35/441	1.26 (0.79–2.02)	0.330	1.22 (0.73–2.04)	0.440	1.20 (0.71–2.01)	0.499
≥8	87/860	2.15 (1.57–2.94)	<0.001	2.12 (1.53–2.94)	<0.001	2.22 (1.51–3.26)	<0.001
Continuous (per 1 h)	197/2,335	1.09 (1.06–1.13)	<0.001	1.09 (1.05–1.13)	<0.001	1.09 (1.05–1.13)	<0.001
Non-CVD mortality							
DST groups (h/days)							
<6	139/1,034	Reference		Reference		Reference	
6–<8	72/441	1.33 (0.83–2.13)	0.234	1.31 (0.83–2.06)	0.251	1.26 (0.81–1.98)	0.307
≥8	144/860	1.69 (1.24–2.30)	0.001	1.64 (1.19–2.27)	0.003	1.75 (1.29–2.36)	<0.001
Continuous (per 1 h)	355/2,335	1.06 (1.02–1.11)	0.003	1.05 (1.01–1.10)	0.012	1.06 (1.02–1.10)	0.004

^a^ Model 1 was adjusted for age (<60, ≥60 years), sex (male, female), and race/ethnicity (non-Hispanic white, others).

^b^ Model 2 was further adjusted for obesity (yes, no), marital status (married, others), family poverty-income ratio (<3.5, ≥3.5), education attainment (above high school, high school and below), current smoking (yes, no), current drinking (yes, no), hypertension (yes, no), diabetes (yes, no), and hypercholesterolemia (yes, no).

CVD, cardiovascular disease; DST, daily sitting time; HR, hazard ratio; LTPA, leisure-time physical activity.

### Joint associations between LTPA and DST and risk of mortality among CVD patients

**Figure 3:**
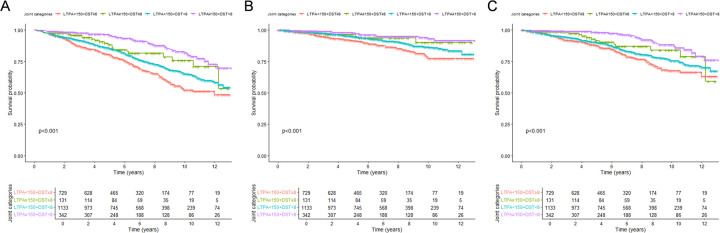
Kaplan-Meier survival curves for mortality, stratified by joint variables of leisure-time physical activity and daily sitting time. **(A)** All-cause mortality; **(B)** CVD mortality; **(C)** non-CVD mortality. DST, daily sitting time; LTPA, leisure-time physical activity.

**Figure 4:**
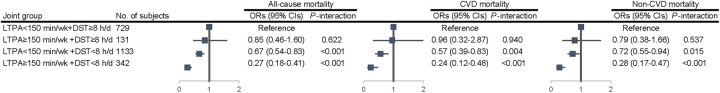
The forest plot of joint analysis.

The survival rates of all-cause, CVD, and non-CVD mortality were significantly different among the four groups (all log-rank tests *P* < 0.001), with the highest survival rates observed for sufficiently active individuals who sat <6 h/days (Figures 3,4). Compared with the referent group (those sitting ≥8 h/days and physically inactive), sufficiently active subjects with sitting <6 h/days were confronted with 73% (HR: 0.27, 95% CI: 0.18–0.41) lower risk of all-cause mortality, 76% (HR: 0.24, 95% CI: 0.12–0.48) lower risk of CVD mortality, and 72% (HR: 0.28, 95% CI: 0.17–0.47) lower risk of non-CVD mortality, respectively (Table 3).

**Table 3:** Joint associations of leisure-time physical activity and daily sitting time with mortality among CVD patients.

Outcome	Event/No.	Crude model		Model 2^b^		Model 1^a^	
		HR (95% CI)	*P*-value	HR (95% CI)	*P*-value	HR (95% CI)	*P*-value
All-cause mortality							
LTPA < 150 min/week + DST ≥ 8 h/days	210/729	Reference		Reference		Reference	
LTPA ≥ 150 min/week + DST ≥ 8 h/days	21/131	0.53 (0.28–0.99)	0.045	0.55 (0.29–1.05)	0.069	0.85 (0.46–1.60)	0.622
LTPA < 150 min/week + DST < 8 h/days	280/1,133	0.69 (0.56–0.85)	0.001	0.73 (0.58–0.93)	0.009	0.67 (0.54–0.83)	<0.001
LTPA ≥ 150 min/week + DST < 8 h/days	41/342	0.22 (0.15–0.34)	<0.001	0.22 (0.14–0.33)	<0.001	0.27 (0.18–0.41)	<0.001
CVD mortality							
LTPA < 150 min/week + DST ≥ 8 h/days	80/729	Reference		Reference		Reference	
LTPA ≥ 150 min/week + DST ≥ 8 h/days	7/131	0.63 (0.23–1.68)	0.352	0.63 (0.24–1.67)	0.355	0.96 (0.32–2.87)	0.940
LTPA < 150 min/week + DST < 8 h/days	95/1,133	0.59 (0.43–0.81)	0.001	0.61 (0.44–0.86)	0.004	0.57 (0.39–0.83)	0.004
LTPA ≥ 150 min/week + DST < 8 h/days	15/342	0.20 (0.10–0.40)	<0.001	0.19 (0.10–0.37)	<0.001	0.24 (0.12–0.48)	<0.001
Non-CVD mortality							
LTPA < 150 min/week + DST ≥ 8 h/days	130/729	Reference		Reference		Reference	
LTPA ≥ 150 min/week + DST ≥ 8 h/days	14/131	0.47 (0.22–1.02)	0.055	0.51 (0.23–1.13)	0.096	0.79 (0.38–1.66)	0.537
LTPA < 150 min/week + DST < 8 h/days	185/1,133	0.75 (0.57–0.98)	0.033	0.80 (0.60–1.07)	0.138	0.72 (0.55–0.94)	0.015
LTPA ≥ 150 min/week + DST < 8 h/days	26/342	0.23 (0.14–0.39)	<0.001	0.23 (0.14–0.39)	<0.001	0.28 (0.17–0.47)	<0.001

^a^ Model 1 was adjusted for age (<60, ≥60 years), sex (male, female), and race/ethnicity (non-Hispanic white, others).

^b^ Model 2 was further adjusted for obesity (yes, no), marital status (married, others), family poverty-income ratio (<3.5, ≥3.5), education attainment (above high school, high school and below), current smoking (yes, no), current drinking (yes, no), hypertension (yes, no), diabetes (yes, no), and hypercholesterolemia (yes, no).

CVD, cardiovascular disease; DST, daily sitting time; HR, hazard ratio; LTPA, leisure-time physical activity.

### Sensitivity analyses

The observed significant associations after excluding early deaths occurring in the first one year of follow-up. Compared with physically inactive individuals, sufficiently active individuals were faced with 11% (HR: 0.89, 95% CI: 0.83–0.96) lower risk of all-cause mortality, 14% (HR: 0.86, 95% CI: 0.77–0.97) lower risk of CVD mortality, and 9% (HR: 0.91, 95% CI: 0.84–0.98) lower risk of non-CVD mortality, respectively (Supplementary Table S9). Compared with those with DST < 6 h/days, subjects with sitting ≥8 h/days were faced with 94% (HR: 1.94, 95% CI: 1.53–2.46) higher risk of all-cause mortality, 135% (HR: 2.35, 95% CI: 1.55–3.54) higher risk of CVD mortality, and 77% (HR: 1.77, 95% CI: 1.31–2.40) higher risk of non-CVD mortality, respectively (Supplementary Table S9). Per hour increase in DST was associated with 7% (HR: 1.07, 95% CI: 1.03–1.10) greater risk of all-cause mortality, 9% (HR: 1.09, 95% CI: 1.05–1.13) greater risk of CVD mortality, and 5% (HR: 1.05, 95% CI: 1.01–1.10) greater risk of non-CVD mortality, respectively (Supplementary Table S9). Moreover, significantly decreased risks of mortality were found in sufficiently active subjects with sitting <6 h/days, compared with the referent group (Supplementary Table S10). Compared with the referent group (those sitting ≥8 h/days and physically inactive), sufficiently active subjects with sitting <6 h/days were confronted with 74% (HR: 0.26, 95% CI: 0.17–0.40) lower risk of all-cause mortality, 80% (HR: 0.20, 95% CI: 0.09–0.44) lower risk of CVD mortality, and 71% (HR: 0.29, 95% CI: 0.17–0.48) lower risk of non-CVD mortality, respectively (Supplementary Table S10). The *E*-values suggested that it would take strong confounding to negate the significant associations observed in our study (Supplementary Table S11).

## Discussion

This prospective cohort study, utilizing a nationally representative sample of US adults with pre-existing CVD, establishes that higher levels of LTPA and lower DST are independently linked to significantly decreased risks of all-cause, CVD, and non-CVD mortality. Notably, the combined analysis indicates that individuals engaging in sufficient activity and sitting for under 6 h daily exhibit the lowest mortality risk, with a substantial reduction exceeding 70% compared to inactive individuals sitting for 8 or more hours daily. These results underscore the crucial roles of increasing physical activity and reducing sedentary time in the secondary prevention of mortality among CVD patients.

The independent association of LTPA with reduced mortality risk in our CVD cohort is consistent with existing evidence in the general population (18–20), as well as in other patient groups such as those with type 2 diabetes (2, 21) and cancer survivors (3, 12). Our finding that each additional hour of weekly LTPA is linked to a 10%–12% decrease in mortality risk highlights the dose-response relationship of physical activity. Even inadequate activity levels (1–<150 min/week) show a potential decrease in risk compared to being entirely inactive, although meeting or exceeding 150 min/week provides the most significant benefits, in line with current physical activity guidelines (22, 23).

Similarly, the associations of prolonged DST observed in our study are well-documented. Higher DST has been associated with elevated all-cause and CVD mortality rates across various populations (24–26). Specifically, in individuals with CVD such as stroke survivors, sitting for ≥8 h per day has been linked to increased mortality risk (13). Our results, indicating a 7% higher risk of all-cause mortality for each additional hour of DST and a 45% increased risk for those sitting ≥8 h per day compared to <6 h per day, emphasize the importance of reducing sedentary time as a critical health goal for CVD patients (8). The average individual may spend a considerable portion of their day in a sedentary state (27), with older adults averaging 9.4 h of sedentary behavior daily (5).

Our study's most significant finding is the combined associations of LTPA and DST on mortality. Individuals engaging in adequate physical activity and maintaining low DST (<6 h/day) demonstrated the lowest mortality risk, indicating a synergistic advantage. This aligns with previous research, such as a study on stroke survivors showing that active individuals sitting <6 h/day had notably lower all-cause mortality risk compared to inactive individuals sitting ≥8 h/day (13). Similarly, research on cancer survivors by Cao (2022) and Yu (2023) found that being physically active and sitting less correlated with improved survival rates (3, 28). Our study expands on these findings to a wider CVD population. However, among those with high DST (≥8 h/day), meeting recommended activity levels did not show a statistically significant mortality reduction compared to being inactive with high DST (all-cause HR: 0.85, 95% CI: 0.46–1.60; CVD HR: 0.96, 95% CI: 0.32–2.87; non-CVD HR: 0.79, 95% CI: 0.38–1.66), though the point estimates suggest a potential protective effect. This finding contrasts with prior evidence, including a comprehensive meta-analysis (29), indicating that adequate physical activity can partially offset the risks of prolonged sedentary behavior. Possible explanations for this discrepancy include the smaller sample size in the high DST and sufficient LTPA subgroup (*n* = 131), leading to wide confidence intervals and reduced statistical power; the specific vulnerability of CVD patients, who may require higher activity thresholds to mitigate sedentary risks; or limitations in self-reported measures, which could introduce misclassification bias. Conversely, among inactive individuals, lower DST was associated with better survival outcomes, highlighting the benefits of reducing sitting time even if activity guidelines are not fully met. Studies on occupational sitting further suggest that high levels of LTPA can mitigate the risks linked to prolonged sitting during work hours (11, 30), which is relevant here as DST in our study encompasses both leisure and occupational sitting time. However, it is essential to acknowledge that occupational physical activity (OPA) may not yield the same cardiovascular health advantages as leisure-time physical activity (31, 32), underscoring why our focus on LTPA is particularly pertinent for CVD patients, where intentional, moderate-to-vigorous activity may be more beneficial than incidental OPA.

Physical activity has diverse effects on cardiovascular health, including enhancing endothelial function, reducing inflammation, improving insulin sensitivity, lowering blood pressure, optimizing lipid profiles, and aiding in weight management (33). In contrast, prolonged sitting is linked to adverse metabolic outcomes such as impaired glucose metabolism, elevated triglycerides, and decreased HDL cholesterol, possibly due to reduced muscle contractile activity and its effects on lipoprotein lipase function (7, 34). Sedentary behavior can also result in increased arterial stiffness and compromised vascular function (27). The combination of regular physical activity and reduced sitting time likely optimizes these physiological pathways, thereby enhancing cardiovascular health and longevity. Moreover, physical activity can influence body fat distribution, which is associated with mortality rates (35–37).

Our study possesses several strengths. Firstly, it utilized data from NHANES, a large, nationally representative sample of US adults, thereby enhancing the generalizability of our findings to the US CVD population. Secondly, the prospective cohort design enabled the examination of temporal relationships between LTPA, DST, and mortality. Thirdly, mortality outcomes were determined through linkage to the NDI, ensuring precise and comprehensive data.

Several limitations need to be acknowledged. Firstly, the self-reported nature of LTPA and DST may introduce recall bias and social desirability bias, potentially leading to misclassification (38). The utilization of objective measures, such as accelerometers, would offer more precise assessments. Secondly, LTPA and DST were only evaluated at baseline, failing to capture changes in these behaviors over the follow-up period, which could impact the associations. Thirdly, despite adjusting for numerous covariates, the possibility of residual confounding by unmeasured factors (e.g., medication adherence, severity of CVD) remains, although E-values indicate substantial robustness. Fourthly, the observational design of the study prevents definitive causal inferences. Fifthly, the definition of CVD relied on self-reported physician diagnosis, introducing a potential misclassification bias.

The implications of our findings for clinical and public health are substantial. For patients with CVD, who face a high risk of subsequent events and mortality, promoting LTPA and reducing DST should be key components of secondary prevention strategies. Healthcare providers should regularly evaluate and advise CVD patients on both behaviors. Interventions should not only focus on structured exercise programs but also on integrating more movement into daily activities and breaking up prolonged periods of sitting. Public health campaigns should stress the importance of “moving more and sitting less” for this vulnerable population. Even for patients struggling to meet recommended LTPA levels, reducing DST can still yield survival benefits. Future clinical intervention studies are required to confirm the findings of this study and offer more robust evidence. Studies incorporating objective measures of activity and sedentary time would be valuable. Furthermore, examining different types and domains of physical activity (e.g., occupational, transport-related) and sedentary behaviors (e.g., TV viewing, computer use) could offer additional insights.

## Conclusion

In conclusion, this study suggests that increasing leisure-time physical activity and reducing sedentary time may have potential benefits for reducing mortality in patients with cardiovascular disease. Future research should incorporate objective measures, such as accelerometers, for assessing LTPA and DST to mitigate self-report biases and validate these associations. Additionally, randomized controlled intervention trials are needed to establish causality, evaluate the long-term effects of targeted programs promoting physical activity and reducing sitting time, and explore the impacts of different domains (e.g., occupational, transport-related) and types (e.g., screen-based vs. non-screen) of these behaviors on mortality outcomes in diverse CVD populations.

## Data Availability

Publicly available datasets were analyzed in this study. This data can be found here: The datasets utilized or examined in this study can be accessed at https://www.cdc.gov/nchs/nhanes/?CDC_AAref_Val=https://www.cdc.gov/nchs/nhanes/index.htm.
