# Supplementary material for: Leisure-time physical activity, daily sitting time, and risk of mortality among CVD patients: a prospective cohort study
Source: Front Cardiovasc Med. 2025 Oct 2;12:1672135. doi: 10.3389/fcvm.2025.1672135 (PMC12528011; doi:10.3389/fcvm.2025.1672135)
Supplement: Supplementary file 1 [file Datasheet1.pdf]

Supplementary Materials

Leisure-Time Physical Activity, Daily Sitting Time, and Risk of Mortality Among CVD Patients: A Prospective Cohort Study From the NHANES

Table S1. Results of proportional hazards assumptions tests. 2

Table S2. Characteristics of the study population according to the levels of daily sitting time. 4

Table S3. Association between leisure-time physical activity and all-cause mortality stratified by confounders. 6

Table S4. Association between leisure-time physical activity and CVD mortality stratified by confounders. 8

Table S5. Association between leisure-time physical activity and non-CVD mortality stratified by confounders. 10

Table S6. Association between daily sitting time and all-cause mortality stratified by confounders. 12

Table S7. Association between daily sitting time and CVD mortality stratified by confounders. 14

Table S8. Association between daily sitting time and non-CVD mortality stratified by confounders. 16

Table S9. The associations of leisure-time physical activity and daily sitting time with all-cause, CVD, and non-CVD mortality among CVD patients after excluding early deaths occurring in the first year of follow-up. 18

Table S10. Joint associations of leisure-time physical activity and daily sitting time with mortality among CVD patients after excluding early deaths occurring in the first year of follow-up. 20

Table S11. E-values and lower limit of 95% CIs for the associations of leisure-time physical activity and daily sitting time with all-cause, CVD, and non-CVD mortality among CVD patients. 21

Figure S1. Flow of eligible participants selection. 22

Table S1. Results of proportional hazards assumptions tests.

Exposure	Outcome	Variable	*P*-value for individual variables	Global *P*-value
LTPA	All-cause mortality	Age	0.93	1.00
		Sex	1.00	
		Race/ethnicity	0.96	
		Obesity	0.96	
		Marital status	0.94	
		Family poverty-income ratio	0.98	
		Education attainment	0.97	
		Current smoking	0.98	
		Current drinking	1.00	
		Hypertension	0.99	
		Diabetes	0.96	
		Hypercholesterolemia	1.00	
	CVD mortality	Age	0.95	1.00
		Sex	0.99	
		Race/ethnicity	0.97	
		Obesity	0.99	
		Marital status	0.98	
		Family poverty-income ratio	0.99	
		Education attainment	0.99	
		Current smoking	0.97	
		Current drinking	0.99	
		Hypertension	1.00	
		Diabetes	1.00	
		Hypercholesterolemia	1.00	
		LTPA	1.00	
	Non-CVD mortality	Age	0.94	1.00
		Sex	0.99	
		Race/ethnicity	0.98	
		Obesity	0.96	
		Marital status	0.94	
		Family poverty-income ratio	0.98	
		Education attainment	0.97	
		Current smoking	0.99	
		Current drinking	0.99	
		Hypertension	0.99	

		Diabetes	0.95	
		Hypercholesterolemia	0.99	
		LTPA	0.96	
DST	All-cause mortality	Age	0.93	1.00
		Sex	0.99	
		Race/ethnicity	0.96	
		Obesity	0.97	
		Marital status	0.94	
		Family poverty-income ratio	0.99	
		Education attainment	0.97	
		Current smoking	0.97	
		Current drinking	1.00	
		Hypertension	0.99	
		Diabetes	0.97	
		Hypercholesterolemia	1.00	
		LTPA	0.96	
	CVD mortality	Age	0.95	1.00
		Sex	0.99	
		Race/ethnicity	0.97	
		Obesity	0.99	
		Marital status	0.98	
		Family poverty-income ratio	0.99	
		Education attainment	0.99	
		Current smoking	0.97	
		Current drinking	0.99	
		Hypertension	1.00	
		Diabetes	1.00	
		Hypercholesterolemia	1.00	
		LTPA	0.98	
	Non-CVD mortality	Age	0.94	1.00
		Sex	0.99	
		Race/ethnicity	0.98	
		Obesity	0.97	
		Marital status	0.94	
		Family poverty-income ratio	0.98	
		Education attainment	0.98	
		Current smoking	0.99	
		Current drinking	0.99	

		Hypertension	0.99	
		Diabetes	0.96	
		Hypercholesterolemia	0.99	
		LTPA	0.97	

Table S2. Characteristics of the study population according to the levels of daily sitting time.

**Variable**[a]	**All (n=2335)**	**Levels of daily sitting time**			
		**<6 (n=1572)**	**6-<8 (n=290)**	**≥8 (n=473)**	***P*-value**[b]
Age, years	61.56 (0.33)	60.30 (0.50)	62.85 (0.60)	62.28 (0.50)	0.003
BMI, kg/m^2	31.55 (0.22)	30.31 (0.28)	31.24 (0.58)	33.02 (0.39)	0.03
Male, n (%)	1361 (57.12)	593 (56.13)	274 (60.49)	494 (56.55)	0.47
Obesity, n (%)	1203 (53.61)	476 (47.10)	233 (54.31)	494 (60.18)	<0.001
Race/ethnicity, n (%)					<0.001
Non-Hispanic white	1114 (71.31)	435 (66.49)	220 (73.05)	459 (75.56)	
Non-Hispanic black	590 (12.61)	253 (12.81)	110 (12.19)	227 (12.60)	
Mexican American	243 (4.76)	149 (7.04)	42 (4.18)	52 (2.61)	
Others	388 (11.33)	197 (13.65)	69 (10.57)	122 (9.23)	
Marital status, n (%)					0.22
Married	1200 (57.09)	544 (60.10)	235 (57.78)	421 (53.56)	
Single	1012 (37.04)	433 (34.11)	181 (35.20)	398 (41.04)	
Living with a partner	123 (5.87)	57 (5.79)	25 (7.03)	41 (5.40)	
Education attainment, n (%)					0.14
Under high school	746 (21.68)	378 (24.85)	129 (20.80)	239 (18.74)	
High school	607 (27.83)	252 (28.10)	125 (29.12)	230 (26.91)	
Above high school	982 (50.49)	404 (47.05)	187 (50.07)	391 (54.35)	
Family PIR, n (%)					0.84
<1.3	980 (29.95)	448 (29.90)	178 (30.83)	354 (29.58)	
1.3-<3.5	881 (39.17)	399 (40.90)	173 (39.52)	309 (37.16)	
≥3.5	474 (30.88)	187 (29.20)	90 (29.64)	197 (33.26)	
Current smoking, n (%)	618 (25.92)	267 (24.42)	129 (31.51)	222 (24.80)	0.13
Current drinking, n (%)	1277 (60.30)	554 (59.42)	246 (59.78)	477 (61.49)	0.74
Hypertension, n (%)	1812 (73.58)	786 (72.41)	352 (74.28)	674 (74.49)	0.78
Diabetes, n (%)	1011 (38.46)	417 (32.72)	178 (36.54)	416 (45.46)	<0.001
Hypercholesterolemia, n (%)	1637 (72.01)	719 (69.87)	309 (74.31)	609 (73.15)	0.26
LTPA, min/wk, n (%)					<0.001
0	1572 (62.07)	635 (54.41)	309 (59.48)	628 (71.44)	
1-<150	290 (13.50)	139 (15.43)	50 (12.74)	101 (11.84)	
≥150	473 (24.42)	260 (30.16)	82 (27.79)	131 (16.72)	

[a] Continuous variables were expressed as weighted means and standard errors and categorical variables were expressed as numbers and weighted percentages. The sums of percentages may not reach 100%, owing to the rounding of decimals.

[b] Characteristics across levels of daily sitting time were compared with linear regression for continuous variables and logistic regression for categorical variables.

Abbreviations: BMI, body mass index; DST, daily sitting time; PIR, poverty-income ratio.

Table S3. Association between leisure-time physical activity and all-cause mortality stratified by confounders.

Characteristics	No. of subjects	HR (95% CI)	*P*-interaction
**Overall**	2335	0.90 (0.85-0.95)	
**Age, years**			0.679
<60	748	0.95 (0.85-1.06)	
≥60	1587	0.89 (0.83-0.95)	
**Sex**			0.088
Male	1361	0.92 (0.86-0.98)	
Female	974	0.80 (0.70-0.92)	
**Race/ethnicity**			0.782
Others	1361	0.92 (0.86-0.98)	
Non-Hispanic white	974	0.80 (0.70-0.92)	
**Obesity**			0.487
No	1132	0.91 (0.85-0.98)	
Yes	1203	0.86 (0.77-0.96)	
**Marital status**			0.961
Others	1135	0.89 (0.83-0.96)	
Married	1200	0.90 (0.82-0.99)	
**Education attainment**			0.179
High school and below	1353	0.93 (0.87-0.99)	
Above high school	982	0.86 (0.76-0.96)	
**Family PIR**			0.239
<3.5	1861	0.88 (0.82-0.94)	
≥3.5	474	0.94 (0.86-1.03)	
**Current smoking**			0.099
No	1717	0.92 (0.86-0.98)	
Yes	618	0.77 (0.64-0.92)	
**Current drinking**			0.397
No	1058	0.91 (0.84-1.00)	
Yes	1277	0.88 (0.83-0.95)	
**Hypertension**			0.506
No	523	0.94 (0.85-1.04)	
Yes	1812	0.88 (0.82-0.95)	
**Diabetes**			0.767
No	1324	0.89 (0.82-0.97)	
Yes	1011	0.90 (0.81-0.99)	
**Hypercholesterolemia**			0.815
No	698	0.90 (0.82-1.00)	
Yes	1637	0.90 (0.83-0.97)	

Models were adjusted for age (<60, ≥60 years), sex (male, female), race/ethnicity (non-Hispanic white,

others), obesity (yes, no), marital status (married, others), family poverty-income ratio (<3.5, ≥3.5), education attainment (above high school, high school and below), current smoking (yes, no), current drinking (yes, no), hypertension (yes, no), diabetes (yes, no), and hypercholesterolemia (yes, no). Abbreviations: HR, hazard ratio; PIR, poverty-income ratio.

Table S4. Association between leisure-time physical activity and CVD mortality stratified by confounders.

Characteristics	No. of subjects	HR (95% CI)	*P*-interaction
**Overall**	2335	0.88 (0.79-0.97)	
**Age, years**			0.645
<60	748	0.87 (0.69-1.08)	
≥60	1587	0.88 (0.78-0.99)	
**Sex**			0.408
Male	1361	0.89 (0.79-0.99)	
Female	974	0.80 (0.62-1.05)	
**Race/ethnicity**			0.414
Others	1361	0.92 (0.85-0.99)	
Non-Hispanic white	974	0.85 (0.72-1.00)	
**Obesity**			0.486
No	1132	0.86 (0.74-1.00)	
Yes	1203	0.91 (0.80-1.03)	
**Marital status**			0.377
Others	1135	0.91 (0.82-1.01)	
Married	1200	0.83 (0.72-0.97)	
**Education attainment**			0.107
High school and below	1353	0.93 (0.84-1.02)	
Above high school	982	0.80 (0.67-0.96)	
**Family PIR**			0.523
<3.5	1861	0.85 (0.75-0.95)	
≥3.5	474	0.93 (0.80-1.07)	
**Current smoking**			0.546
No	1717	0.89 (0.79-1.00)	
Yes	618	0.77 (0.55-1.07)	
**Current drinking**			0.353
No	1058	0.90 (0.79-1.03)	
Yes	1277	0.82 (0.72-0.95)	
**Hypertension**			0.102
No	523	0.72 (0.52-1.00)	
Yes	1812	0.90 (0.81-1.00)	
**Diabetes**			0.270
No	1324	0.82 (0.71-0.95)	
Yes	1011	0.92 (0.82-1.04)	
**Hypercholesterolemia**			0.298
No	698	0.81 (0.64-1.01)	
Yes	1637	0.90 (0.82-1.00)	

Models were adjusted for age (<60, ≥60 years), sex (male, female), race/ethnicity (non-Hispanic white,

others), obesity (yes, no), marital status (married, others), family poverty-income ratio (<3.5, ≥3.5), education attainment (above high school, high school and below), current smoking (yes, no), current drinking (yes, no), hypertension (yes, no), diabetes (yes, no), and hypercholesterolemia (yes, no). Abbreviations: HR, hazard ratio; PIR, poverty-income ratio.

Table S5. Association between leisure-time physical activity and non-CVD mortality stratified by confounders.

Characteristics	No. of subjects	HR (95% CI)	*P*-interaction
**Overall**	2335	0.91 (0.84-0.98)	
**Age, years**			0.557
<60	748	0.97 (0.87-1.07)	
≥60	1587	0.89 (0.82-0.97)	
**Sex**			0.108
Male	1361	0.94 (0.87-1.01)	
Female	974	0.80 (0.68-0.93)	
**Race/ethnicity**			0.811
Others	1361	0.88 (0.80-0.97)	
Non-Hispanic white	974	0.91 (0.84-0.99)	
**Obesity**			0.230
No	1132	0.93 (0.87-1.00)	
Yes	1203	0.82 (0.70-0.97)	
**Marital status**			0.756
Others	1135	0.88 (0.79-0.98)	
Married	1200	0.93 (0.84-1.02)	
**Education attainment**			0.417
High school and below	1353	0.93 (0.85-1.01)	
Above high school	982	0.88 (0.77-1.00)	
**Family PIR**			0.296
<3.5	1861	0.89 (0.81-0.97)	
≥3.5	474	0.95 (0.84-1.07)	
**Current smoking**			0.072
No	1717	0.93 (0.87-1.00)	
Yes	618	0.76 (0.63-0.91)	
**Current drinking**			0.635
No	1058	0.92 (0.81-1.04)	
Yes	1277	0.90 (0.84-0.97)	
**Hypertension**			0.117
No	523	0.98 (0.90-1.07)	
Yes	1812	0.87 (0.80-0.95)	
**Diabetes**			0.340
No	1324	0.91 (0.84-1.00)	
Yes	1011	0.87 (0.76-1.00)	
**Hypercholesterolemia**			0.518
No	698	0.93 (0.84-1.02)	
Yes	1637	0.90 (0.80-1.00)	

Models were adjusted for age (<60, ≥60 years), sex (male, female), race/ethnicity (non-Hispanic white,

others), obesity (yes, no), marital status (married, others), family poverty-income ratio (<3.5, ≥3.5), education attainment (above high school, high school and below), current smoking (yes, no), current drinking (yes, no), hypertension (yes, no), diabetes (yes, no), and hypercholesterolemia (yes, no). Abbreviations: HR, hazard ratio; PIR, poverty-income ratio.

Table S6. Association between daily sitting time and all-cause mortality stratified by confounders.

**Characteristics**	**No. of subjects**	**HR (95% CI)**	***P*-interaction**
**Overall**	2335	1.07 (1.04-1.11)	
**Age, years**			0.257
<60	748	1.08 (1.02-1.16)	
≥60	1587	1.07 (1.03-1.11)	
**Sex**			0.297
Male	1361	1.09 (1.05-1.13)	
Female	974	1.06 (1.00-1.11)	
**Race/ethnicity**			0.327
Others	1361	1.06 (1.02-1.09)	
Non-Hispanic white	974	1.08 (1.04-1.13)	
**Obesity**			0.360
No	1132	1.06 (1.02-1.11)	
Yes	1203	1.09 (1.04-1.13)	
**Marital status**			0.929
Others	1135	1.07 (1.03-1.12)	
Married	1200	1.07 (1.03-1.12)	
**Education attainment**			0.856
High school and below	1353	1.07 (1.03-1.11)	
Above high school	982	1.08 (1.02-1.15)	
**Family PIR**			0.773
<3.5	1861	1.07 (1.03-1.11)	
≥3.5	474	1.10 (1.00-1.21)	
**Current smoking**			0.592
No	1717	1.08 (1.04-1.12)	
Yes	618	1.07 (1.01-1.13)	
**Current drinking**			0.584
No	1058	1.06 (1.02-1.10)	
Yes	1277	1.08 (1.03-1.13)	
**Hypertension**			0.335
No	523	1.04 (0.97-1.11)	
Yes	1812	1.08 (1.05-1.12)	
**Diabetes**			0.749
No	1324	1.06 (1.01-1.12)	
Yes	1011	1.08 (1.04-1.13)	
**Hypercholesterolemia**			0.455
No	698	1.04 (0.98-1.09)	
Yes	1637	1.08 (1.04-1.13)	

Models were adjusted for age (<60, ≥60 years), sex (male, female), race/ethnicity (non-Hispanic white, others), obesity (yes, no), marital status (married, others), family poverty-income ratio (<3.5, ≥3.5),

education attainment (above high school, high school and below), current smoking (yes, no), current drinking (yes, no), hypertension (yes, no), diabetes (yes, no), and hypercholesterolemia (yes, no). Abbreviations: HR, hazard ratio; PIR, poverty-income ratio.

Table S7. Association between daily sitting time and CVD mortality stratified by confounders.

Characteristics	No. of subjects	HR (95% CI)	*P*-interaction
**Overall**	2335	1.09 (1.05-1.13)	
**Age, years**			0.157
<60	748	1.12 (1.01-1.25)	
≥60	1587	1.08 (1.04-1.13)	
**Sex**			0.426
Male	1361	1.11 (1.06-1.16)	
Female	974	1.06 (0.99-1.14)	
**Race/ethnicity**			0.824
Others	1361	1.10 (1.04-1.17)	
Non-Hispanic white	974	1.10 (1.04-1.16)	
**Obesity**			0.451
No	1132	1.11 (1.05-1.18)	
Yes	1203	1.08 (1.02-1.14)	
**Marital status**			0.878
Others	1135	1.09 (1.03-1.15)	
Married	1200	1.09 (1.03-1.16)	
**Education attainment**			0.052
High school and below	1353	1.07 (1.02-1.12)	
Above high school	982	1.16 (1.07-1.25)	
**Family PIR**			0.558
<3.5	1861	1.08 (1.04-1.13)	
≥3.5	474	1.13 (1.00-1.27)	
**Current smoking**			0.643
No	1717	1.08 (1.04-1.13)	
Yes	618	1.14 (1.04-1.26)	
**Current drinking**			0.385
No	1058	1.10 (1.04-1.16)	
Yes	1277	1.07 (1.01-1.14)	
**Hypertension**			0.556
No	523	1.14 (1.02-1.28)	
Yes	1812	1.08 (1.04-1.13)	
**Diabetes**			0.875
No	1324	1.10 (1.03-1.18)	
Yes	1011	1.10 (1.03-1.17)	
**Hypercholesterolemia**			0.983
No	698	1.08 (1.01-1.15)	
Yes	1637	1.10 (1.04-1.15)	

Models were adjusted for age (<60, ≥60 years), sex (male, female), race/ethnicity (non-Hispanic white, others), obesity (yes, no), marital status (married, others), family poverty-income ratio (<3.5, ≥3.5),

education attainment (above high school, high school and below), current smoking (yes, no), current drinking (yes, no), hypertension (yes, no), diabetes (yes, no), and hypercholesterolemia (yes, no). Abbreviations: HR, hazard ratio; PIR, poverty-income ratio.

Table S8. Association between daily sitting time and non-CVD mortality stratified by confounders.

Characteristics	No. of subjects	HR (95% CI)	*P*-interaction
**Overall**	2335	1.06 (1.02-1.10)	
**Age, years**			0.554
<60	748	1.06 (0.98-1.15)	
≥60	1587	1.06 (1.01-1.11)	
**Sex**			0.434
Male	1361	1.08 (1.03-1.14)	
Female	974	1.05 (0.99-1.12)	
**Race/ethnicity**			0.168
Others	1361	1.03 (0.98-1.07)	
Non-Hispanic white	974	1.07 (1.02-1.13)	
**Obesity**			0.121
No	1132	1.04 (0.99-1.10)	
Yes	1203	1.09 (1.04-1.15)	
**Marital status**			0.959
Others	1135	1.06 (1.01-1.12)	
Married	1200	1.07 (1.00-1.13)	
**Education attainment**			0.362
High school and below	1353	1.07 (1.02-1.13)	
Above high school	982	1.04 (0.97-1.12)	
**Family PIR**			0.939
<3.5	1861	1.06 (1.02-1.11)	
≥3.5	474	1.08 (0.96-1.21)	
**Current smoking**			0.372
No	1717	1.08 (1.03-1.13)	
Yes	618	1.04 (0.97-1.10)	
**Current drinking**			0.243
No	1058	1.04 (0.99-1.10)	
Yes	1277	1.08 (1.02-1.14)	
**Hypertension**			0.091
No	523	0.99 (0.92-1.07)	
Yes	1812	1.08 (1.04-1.13)	
**Diabetes**			0.702
No	1324	1.05 (0.99-1.11)	
Yes	1011	1.07 (1.02-1.13)	
**Hypercholesterolemia**			0.397
No	698	1.02 (0.95-1.09)	
Yes	1637	1.08 (1.02-1.13)	

Models were adjusted for age (<60, ≥60 years), sex (male, female), race/ethnicity (non-Hispanic white, others), obesity (yes, no), marital status (married, others), family poverty-income ratio (<3.5, ≥3.5),

education attainment (above high school, high school and below), current smoking (yes, no), current drinking (yes, no), hypertension (yes, no), diabetes (yes, no), and hypercholesterolemia (yes, no). Abbreviations: HR, hazard ratio; PIR, poverty-income ratio.

Table S9. The associations of leisure-time physical activity and daily sitting time with all-cause, CVD, and non-CVD mortality among CVD patients after excluding early deaths occurring in the first year of follow-up.

Outcome	Event/No.	Crude model		Model 1[a]		Model 2[b]	
		HR (95% CI)	*P*-value	HR (95% CI)	*P*-value	HR (95% CI)	*P*-value
**Leisure-time physical activity**							
All-cause mortality							
LTPA groups (min/wk)							
0	385/1523	Reference		Reference		Reference	
1-<150	53/287	0.67 (0.50-0.90)	0.009	0.60 (0.44-0.81)	0.001	0.77 (0.57-1.06)	0.109
≥150	55/465	0.34 (0.23-0.50)	<0.001	0.31 (0.21-0.47)	<0.001	0.44 (0.29-0.65)	<0.001
Continuous (per 1 hour)	493/2275	0.86 (0.79-0.92)	<0.001	0.84 (0.77-0.91)	<0.001	0.89 (0.83-0.96)	0.001
CVD mortality							
LTPA groups (min/wk)							
0	140/1523	Reference		Reference		Reference	
1-<150	16/287	0.73 (0.43-1.25)	0.253	0.66 (0.38-1.15)	0.143	0.86 (0.50-1.49)	0.593
≥150	19/465	0.36 (0.19-0.68)	0.002	0.32 (0.17-0.63)	0.001	0.45 (0.22-0.92)	0.028
Continuous (per 1 hour)	175/2275	0.83 (0.74-0.93)	0.001	0.81 (0.72-0.92)	0.001	0.86 (0.77-0.97)	0.014
Non-CVD mortality							
LTPA groups (min/wk)							
0	245/1523	Reference		Reference		Reference	
1-<150	37/287	0.64 (0.46-0.89)	0.008	0.56 (0.40-0.79)	0.001	0.73 (0.51-1.04)	0.077
≥150	36/465	0.33 (0.20-0.53)	<0.001	0.31 (0.19-0.50)	<0.001	0.43 (0.27-0.68)	<0.001
Continuous (per 1 hour)	318/2275	0.87 (0.79-0.95)	0.002	0.85 (0.77-0.95)	0.002	0.91 (0.84-0.98)	0.016
**Daily sitting time**							
All-cause mortality							
DST groups (h/d)							
<6	192/1011	Reference		Reference		Reference	

6-<8	94/428	1.27 (0.92-1.74)	0.146	1.24 (0.90-1.70)	0.185	1.21 (0.90-1.63)	0.202
≥8	207/836	1.86 (1.47-2.35)	<0.001	1.81 (1.41-2.33)	<0.001	1.94 (1.53-2.46)	<0.001
Continuous (per 1 hour)	493/2275	1.07 (1.03-1.10)	<0.001	1.06 (1.03-1.10)	0.001	1.07 (1.03-1.10)	<0.001
CVD mortality							
DST groups (h/d)							
<6	65/1011	Reference		Reference		Reference	
6-<8	32/428	1.31 (0.80-2.16)	0.280	1.27 (0.74-2.19)	0.378	1.26 (0.72-2.20)	0.416
≥8	78/836	2.25 (1.61-3.16)	<0.001	2.21 (1.56-3.14)	<0.001	2.35 (1.55-3.54)	<0.001
Continuous (per 1 hour)	175/2275	1.09 (1.05-1.13)	<0.001	1.09 (1.05-1.13)	<0.001	1.09 (1.05-1.13)	<0.001
Non-CVD mortality							
DST groups (h/d)							
<6	127/1011	Reference		Reference		Reference	
6-<8	62/428	1.25 (0.79-1.98)	0.349	1.22 (0.78-1.91)	0.379	1.19 (0.77-1.86)	0.432
≥8	129/836	1.69 (1.24-2.30)	0.001	1.63 (1.18-2.27)	0.003	1.77 (1.31-2.40)	<0.001
Continuous (per 1 hour)	318/2275	1.06 (1.01-1.10)	0.008	1.05 (1.00-1.09)	0.032	1.05 (1.01-1.10)	0.008

[a] Model 1 was adjusted for age (<60, ≥60 years), sex (male, female), and race/ethnicity (non-Hispanic white, others).

[b] Model 2 was further adjusted for obesity (yes, no), marital status (married, others), family poverty-income ratio (<3.5, ≥3.5), education attainment (above high school, high school and below), current smoking (yes, no), current drinking (yes, no), hypertension (yes, no), diabetes (yes, no), and hypercholesterolemia (yes, no).

Abbreviation: CVD, cardiovascular disease; DST, daily sitting time; HR, hazard ratio; LTPA, leisure-time physical activity.

Table S10. Joint associations of leisure-time physical activity and daily sitting time with mortality among CVD patients after excluding early deaths occurring in the first year of follow-up.

**Outcome**	**Event/No.**	**Crude model**		**Model 1**[a]		**Model 2**[b]	
		**HR (95% CI)**	***P*-value**	**HR (95% CI)**	***P*-value**	**HR (95% CI)**	***P*-value**
**All-cause mortality**							
LTPA<150 min/wk+DST≥8 h/d	189/708	Reference		Reference		Reference	
LTPA≥150 min/wk +DST≥8 h/d	18/128	0.50 (0.25-0.99)	0.046	0.53 (0.26-1.06)	0.073	0.79 (0.40-1.57)	0.501
LTPA<150 min/wk +DST<8 h/d	249/1102	0.67 (0.54-0.83)	<0.001	0.72 (0.56-0.92)	0.008	0.64 (0.51-0.81)	<0.001
LTPA≥150 min/wk +DST<8 h/d	37/337	0.22 (0.14-0.34)	<0.001	0.21 (0.14-0.33)	<0.001	0.26 (0.17-0.40)	<0.001
**CVD mortality**							
LTPA<150 min/wk +DST≥8 h/d	72/708	Reference		Reference		Reference	
LTPA≥150 min/wk +DST≥8 h/d	6/128	0.59 (0.20-1.72)	0.333	0.60 (0.21-1.75)	0.352	0.87 (0.27-2.84)	0.823
LTPA<150 min/wk +DST<8 h/d	84/1102	0.57 (0.41-0.79)	0.001	0.61 (0.43-0.85)	0.004	0.55 (0.38-0.81)	0.003
LTPA≥150 min/wk +DST<8 h/d	13/337	0.18 (0.08-0.37)	<0.001	0.17 (0.08-0.35)	<0.001	0.20 (0.09-0.44)	<0.001
**Non-CVD mortality**							
LTPA<150 min/wk +DST≥8 h/d	117/708	Reference		Reference		Reference	
LTPA≥150 min/wk +DST≥8 h/d	12/128	0.45 (0.19-1.04)	0.063	0.49 (0.20-1.17)	0.106	0.73 (0.31-1.72)	0.475
LTPA<150 min/wk +DST<8 h/d	165/1102	0.72 (0.54-0.96)	0.024	0.78 (0.57-1.07)	0.119	0.69 (0.52-0.92)	0.010
LTPA≥150 min/wk +DST<8 h/d	24/337	0.24 (0.15-0.41)	<0.001	0.24 (0.14-0.41)	<0.001	0.29 (0.17-0.48)	<0.001

[a] Model 1 was adjusted for age (<60, ≥60 years), sex (male, female), and race/ethnicity (non-Hispanic white, others).

[b] Model 2 was further adjusted for obesity (yes, no), marital status (married, others), family poverty-income ratio (<3.5, ≥3.5), education attainment (above high school, high school and below), current smoking (yes, no), current drinking (yes, no), hypertension (yes, no), diabetes (yes, no), and hypercholesterolemia (yes, no).

Abbreviation: CVD, cardiovascular disease; DST, daily sitting time; HR, hazard ratio; LTPA, leisure-time physical activity.

Table S11. E-values and lower limit of 95% CIs for the associations of leisure-time physical activity and daily sitting time with all-cause, CVD, and non-CVD mortality among CVD patients.

Outcome	Variable	HR (95% CI)	*E*-value
All-cause mortality	LTPA (≥150 min/wk vs 0 min/wk)	0.45 (0.31-0.66)	3.87
	LTPA (per 1 hour)	0.90 (0.85-0.95)	1.46
	DST (≥8 h/d vs <6 h/d)	1.89 (1.50-2.37)	3.19
	DST (per 1 hour)	1.07 (1.04-1.11)	1.34
CVD mortality	LTPA (≥150 min/wk vs 0 min/wk)	0.51 (0.25-1.02)	3.33
	LTPA (per 1 hour)	0.88 (0.79-0.97)	1.53
	DST (≥8 h/d vs <6 h/d)	2.22 (1.51-3.26)	3.87
	DST (per 1 hour)	1.09 (1.05-1.13)	1.40
Non-CVD mortality	LTPA (≥150 min/wk vs 0 min/wk)	0.43 (0.28-0.65)	4.08
	LTPA (per 1 hour)	0.91 (0.84-0.98)	1.43
	DST (≥8 h/d vs <6 h/d)	1.75 (1.29-2.36)	2.90
	DST (per 1 hour)	1.06 (1.02-1.10)	1.31

Abbreviation: CVD, cardiovascular disease; DST, daily sitting time; HR, hazard ratio; LTPA, leisure-time physical activity.

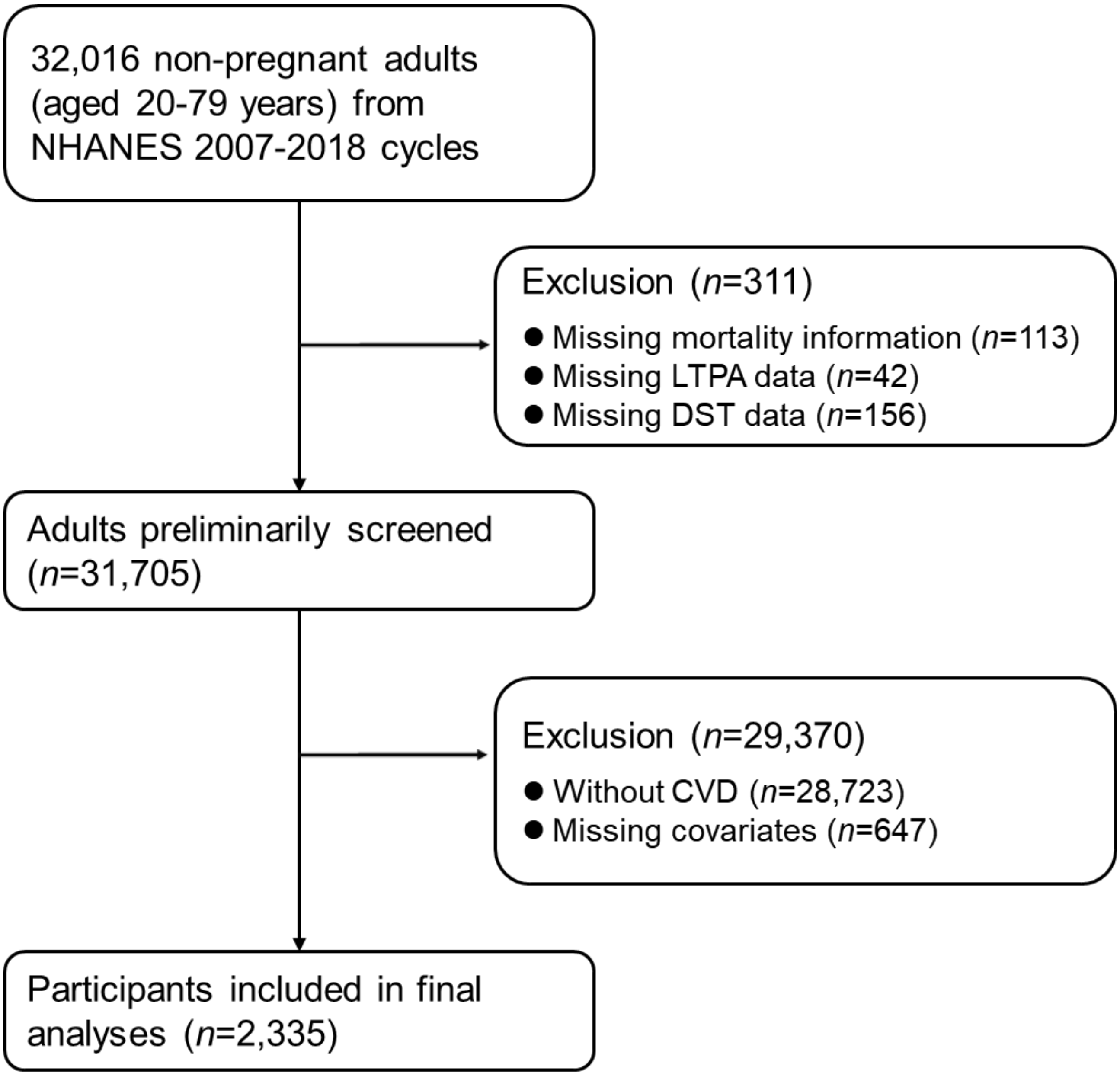

Figure S1. Flow of eligible participants selection.

Abbreviations: CVD, cardiovascular disease; DST, daily sitting time; LTPA, leisure-time physical activity; NHANES, National Health and Nutrition Examination Survey.
